# Outcome of HIV-associated lymphoma in a resource-limited setting of Jos, Nigeria

**DOI:** 10.1186/s13027-017-0144-7

**Published:** 2017-06-05

**Authors:** Olugbenga Akindele Silas, Chad J. Achenbach, Lifang Hou, Robert L. Murphy, Julie O. Egesie, Solomon A. Sagay, Oche O. Agbaji, Patricia E. Agaba, Jonah Musa, Agabus N. Manasseh, Ezra D. Jatau, Ayuba M. Dauda, Maxwell O. Akanbi, Barnabas M. Mandong

**Affiliations:** 1 Pathology Department, Faculty of Medical Sciences, University of Jos/Jos University Teaching Hospital, Jos, Plateau State Nigeria; 2 Feinberg School of Medicine, Department of Medicine, Northwestern University and Center for Global Health, Chicago, Illinois USA; 3 Hematology Department Faculty of Medical Sciences, University of Jos/Jos University Teaching Hospital, Jos, Plateau State Nigeria; 4 Department of Obstetrics and Gynecology Faculty of Medical Sciences, University of Jos/Jos University Teaching Hospital, Jos, Plateau State Nigeria; 5 Department of Internal Medicine Faculty of Medical Sciences, University of Jos/Jos University Teaching Hospital, Jos, Plateau State Nigeria; 6 Department of Family Medicine Faculty of Medical Sciences, University of Jos/Jos University Teaching Hospital, Jos, Plateau State Nigeria

**Keywords:** Human immunodeficiency virus, Mortality, Lymphoma, Outcomes, Nigeria

## Abstract

**Background:**

Lymphoma is a leading cause of cancer-related death among human immunodeficiency virus (HIV)-infected individuals in the current era of potent anti-retroviral therapy (ART). Globally, mortality after HIV-associated lymphoma has profound regional variation. Little is known about HIV-associated lymphoma mortality in Nigeria and other resource-limited setting in sub-Saharan Africa. Therefore, we evaluated the all-cause mortality after lymphoma and associated risk factors including HIV at the Jos University Teaching Hospital (JUTH) Nigeria.

**Methods:**

We conducted a ten-year retrospective cohort study of lymphoma patients managed in JUTH. The main outcome measured was all-cause mortality and HIV infection was the main exposure variable. Overall death rate was estimated using the total number of death events and cumulative follow up time from lymphoma diagnosis to death. Cox proportional hazard regression was used to assess factors associated with mortality after lymphoma diagnosis.

**Results:**

Out of 40 lymphoma patients evaluated, 8(20.0%) were HIV positive and 32(80.0%) were HIV negative. After 127.63 person- years of follow-up, there were 16 deaths leading to a crude mortality rate of 40.0 per 100 person-years. The 2-year probability of survival was 30% for HIV-infected patients and 74% for HIV-uninfected. Median survival probability for HIV-infected patients was 2.1 years and 7.6 years for those without HIV. Unadjusted hazard of death was associated with late stage, HR 11.33(95% CI 2.55, 50.26,*p* = 0.001); low cumulative cycles of chemotherapy, HR 6.43(95% CI 1.80, 22.89,*p* = 0.004); greater age, HR 5.12(95% CI 1.45,18.08,*p* = 0.01); presence of comorbidity, HR 3.43(95% CI 1.10,10.78,*p* = 0.03); and HIV-infection, HR 3.32(95% CI 1.05, 10.51,*p* = 0.04). In an adjusted model only stage was significantly associated with death, AHR 5.45(1.14–26.06, *p* = 0.03).

**Conclusion:**

Our findings suggest that HIV- infection accounted for three times probability of death in lymphoma patients compared to their HIV-uninfected counterparts due to late stage of lymphoma presentation in this population. Also initiation of chemotherapy was associated with lower probability of death among lymphoma patients managed at JUTH, Nigeria. Earlier stage at lymphoma diagnosis and prompt therapeutic intervention is likely to improve survival in these patients. Future research should undertake collaborative studies to obtain comprehensive regional data and identify unique risk factors of poor outcomes among HIV-infected patients with lymphoma in Nigeria.

## Background

Lymphoma accounts for approximately 3% (non-Hodgkin) and 0.5% (Hodgkin) of all cancers worldwide [1–3]. HIV-infected individuals experience lymphoma rates 5–15 times higher than those without HIV, even in the modern era of potent antiretroviral therapy (ART) [4–8]. According to global information and advice on HIV/AIDS (AVERT) 2015, of all people living with HIV globally 9% live in Nigeria and account for 14% of global AIDS-related deaths [3]. In Nigeria, the prevalence of HIV-infected individuals diagnosed with lymphomas increased from 2.3% in 2009 to 4.3% in 2010 [8].

Survival outcomes reported for HIV-lymphomas worldwide are conflicting and few. While a few studies state equal health outcomes comparable to HIV-uninfected lymphoma patients, most studies report poor 2 years survival outcomes with wide ranges between 24.4 and 71.7% for HIV-infected lymphoma from the US and Europe. This variability in mortality outcomes could be due to differences in demographic or disease factors such as age, stage, co-morbidities or histology [7–30]. The effect of disease specific factors such as stage of lymphoma, presence of cumulative number of cycles of chemotherapy received and proper use of antiretroviral (ART) drug use in this setting are unclear [31–33]. Thus this study aims to assess the contributory factors to mortality unique to Nigeria.

## Methods

### Study setting and design

We conducted a 10-year retrospective cohort study from January 1, 2005 to March 31, 2015 of adult lymphoma patients seen at the Jos University Teaching Hospital (JUTH) in Jos, north central Nigeria. Patients were followed up to determine their patient time contribution from the beginning of the study (time of initiation of chemotherapy for lymphoma) to occurrence of the primary outcome (death) or the end of study period. Those lost to follow-up were censored at data of their last known follow-up in clinic.

### Inclusion criteria

We included all adult (18 ± years of age) patients diagnosed with lymphoma and managed at JUTH from January 1, 2005 to March 31, 2015.

### Exclusion criteria

We excluded adults with treatment, follow-up, and care for lymphoma obtained outside of JUTH. We also excluded lymphoma patients who were pregnant.

### Data collection

Demographic variables (age, sex), clinical variables (comorbidity, HIV sero-status, stage, subtype, chemotherapy cycles), laboratory variables (CD4+ T cell count, HIV RNA level), follow up and outcome(mortality) data for the HIV-positive lymphoma group were obtained from the electronic records of the AIDS Prevention Initiative in Nigeria (APIN) center of JUTH. Data for the HIV-negative group were obtained from the case report forms from the Hematology department and electronic database of the Pathology department of JUTH. Other sources of data were the inpatient notes, hospital discharge summaries, mortuary records, and from phone calls to patients or their relatives.

#### Demographic variables measured

Sex was either male or female, age was analyzed as continuous variable.

#### Clinical variables measured

The primary outcome variable was all-cause mortality (determined as below), and the main exposure variable was HIV infection (by HIV antibody testing). Other variables measured were cumulative cycles of chemotherapy received within a year after lymphoma diagnosis, baseline CD4+ T cell count, baseline HIV RNA level, comorbidities, tumor stage, histologic tumor type and ART use. Baseline nadir CD4+ T cell count was the lowest value before or at initiation of ART and baseline HIV RNA level was the peak level before or at initiation of ART. Information on mortality was obtained from hospital records, mortuary death records and telephone interview with family of patient. Histologic tumor type was based on hematoxylin and eosin stain microscopic pathology report and divided into non-Hodgkin lymphoma (NHL) and Hodgkin lymphoma (HL) according to the W.H.O 2008 classification of lymphoma [Appendix B]. Tumor stage at lymphoma diagnosis was according to Ann Arbor staging with stages I and II (early stage), stages III and IV (late stage) [Appendix A]. Time from lymphoma diagnosis to death was calculated in days and subsequently converted to lunar months and years. Comorbidities were other illnesses recorded for the patients other than lymphoma and HIV infection. Cumulative cycles of chemotherapy was calculated as the total cycles of chemotherapy received by each patient within the first year after lymphoma diagnosis. Patients were considered to be on ART if they had been receiving any combination ART at least 3 months before initiation of lymphoma chemotherapy. Clinically relevant cut-off levels for binary variables CD4+ T cell count and HIV RNA levels were 200 cell/μl and 400 copies/ml respectively.

### Statistical analysis

The analytical focus of this study was to determine all-cause probability of death and to identify likely risk factors that contribute to death in lymphoma patients in this low resource setting. The overall mortality rate was estimated using total number of death events and the cumulative person time follow-up from initiation of chemotherapy to death. Differences in proportions and means between the HIV-infected and HIV-uninfected patients was assessed using Chi-square or Fisher’s exact tests for proportions and t-test for mean. Mean and standard deviations for baseline HIV RNA levels were log transformed for easy interpretation. Cox proportional hazard analysis (unadjusted) was used to examine the association between all-cause mortality in the lymphoma patients and variables age, sex, stage, histologic subtype, cumulative cycles of chemotherapy used, comorbidity and HIV infection. Adjusted analysis was then conducted. *P* value <0.05 was considered significant and only those having significant *p* values were used in the adjusted model. A further analysis was done for the HIV-associated lymphoma group using Chi-square or Fisher’s where applicable for categorical variables and student t-test for means of continuous variables. All statistical tests were 2-sided with type 1 error set at 0.05 for statistical significance. Sex, age, cumulative cycles of chemotherapy, HIV-status, comorbidities, stage, histologic tumor subtype, baseline CD4+ T cell count and baseline HIV RNA level were all treated as binary variables for determining predictors of death and for the further analysis of the HIV group respectively. To estimate the hazard of death following lymphoma diagnosis, we used time from diagnosis in years as time covariate and death as failure event. Kaplan-Meier graph estimating probability of survival following lymphoma diagnosis by HIV status was plotted. We used Log-rank test to test for difference in probability of survival between the HIV positive and HIV negative lymphoma groups with *p*-value 0.05 signifying significant difference in survival. Statistical analysis was performed with STATA version 11.0 college station, Texas, USA.

## Results

### Demographic data

Forty lymphoma patients were identified and followed for a total of 127.63 person-years. Participants comprised 15(37.5%) women and 25(62.5%) men [Table 1].

**Table 1:** A bivariate analysis of demographic/clinical characteristics of lymphoma patients seen at Jos University Teaching Hospital from 01/01/2005 to 03/30/2015 by HIV status

	HIV Negative N (%)	HIV Positive N (%)	Overall N (%)	*P*-value
Number	32 (80.0)	8 (20.0)	40 (100.0)	
Gender, n (%)				
Male	19 (59.4)	6 (75.0)	25 (62.5)	0.41
Female	13 (40.6)	2 (25.0)	15 (37.5)	
Age(yrs)				
≤ 40	18 (56.3)	2 (25.0)	20 (50.0)	0.11
> 40	14 (43.8)	6 (75.0)	20 (50.0)	
Age (Mean, SD) yrs	39.78,15.27	46.38,11.46		0.20
Histologic sub-type				
NHL	28 (87.5)	7 (87.5)	35 (87.5)	1.00
HL	4 (12.5)	1 (12.5)	5 (12.5)	
Stage				
I & II	22 (68.8)	2 (25.0)	24 (60.0)	0.02
III & IV	10 (31.3)	6 (75.0)	16 (40.0)	
Chemotherapy cycles				
> 4	21 (65.6)	3 (37.5)	24 (60.0)	0.15
≤ 4	11 (34.4)	5 (62.5)	16 (40.0)	
Comorbidity				
Absent	18 (56.3)	6 (75.0)	24 (60.0)	0.33
Present	14 (43.8)	2 (25.0)	16 (40.0)	
Mortality (death)				
No deaths	21 (65.6)	3 (37.5)	24 (60.0)	0.15
Deaths	11 (34.4)	5 (62.5)	16 (40.0)	
Total Person years follow up	10.85	116.78	127.63	
Number of loss to Follow up	2	0		

*NHL* non-Hodgkin lymphoma, *HIV* human Immune deficiency virus; Comorbidity e.g. hepatitis, tuberculosis, other cancers. Deaths (all-cause mortality); no death (loss to follow up/alive at end of study)

### Clinical data

Non-Hodgkin lymphoma was the dominant histologic subtype (87.5%). Of the 8 HIV-positive patients, 6(75.0%) presented with late stages III and IV lesions while of the 32 HIV- negative patients 22(68.8%) presented with early stages I and II lesions. Also, the majority of patients (60.0%) received more than four cumulative cycles of chemotherapy. Only 16 (40.0%) patients presented with additional comorbid disease, *p* = 0.33. Comorbidities were mainly hepatitis B, hepatitis C and Kaposi sarcoma. Overall 16/40 (40.0%) of the lymphoma patients died. A total of 2/40 (5.0%) of the lymphoma patients were lost to follow up during the period of study and they were all HIV negative. A total of 8/40 (20.0%) patients were HIV positive and 32/40 (80.0%) were HIV negative. Among those with HIV-associated lymphoma, 75% were male, 6/8 (75%) had late stage presentation (stage III & IV) and 5/8 (62.5%) died. Mean age of HIV-positive patients was higher at 46 years compared to 40 years for the HIV-negative. No other factors were associated with HIV status [Table 1].

Eighty percent of the HIV-associated lymphoma patients who died had baseline CD4+ T cell counts ≤200 cells/μl (*p* = 0.03).

### Unadjusted Cox model

In the unadjusted model, mortality was associated with higher age, HR 5.12 (*p* = 0.01, CI 1.45–18.08), late stage, HR 11.33 (*p* = 0.001, CI 2.55–50.26), low cumulative cycles of chemotherapy, HR 6.43(*p* = 0.004, CI 1.80–22.89), presence of comorbidities, HR 3.43(*p* = 0.03, CI 1.10–10.78) and HIV-infection, HR 3.32(*p* = 0.04, CI 1.05–10.51) [Table 2].

**Table 2:** Unadjusted and Adjusted analysis of characteristics of lymphoma patients using cox- proportional hazards

	Unadjusted model HR (96% CI)	*P*-Value	Adjusted model HR (95% CI)	*P*-Value
Gender				
Male(referent)		0.46		
Female	1.49 (0.51–4.39)			
Age(years)				
≤ 40(referent)				0.16
> 40	5.12 (1.45–18.08)	0.01	1.04 (0.98–1.10)	
Histologic subtype				
HL(referent)		0.99		
NHL	1.01 (0.23–4.52)			
Stage				
I & II(referent)		0.001		0.03
III & IV	11.33 (2.55–50.26)		5.45 (1.14–26.06)	
Chemotherapy cycles				
> 4(referent)		0.004		0.21
≤ 4	6.43 (1.80–22.89)		2.40 (0.61–9.25)	
Comorbidity				
Absent(referent)		0.03		
Present	3.43 (1.10–10.78)			
HIV serostatus				
Negative(referent)		0.04		0.56
Positive	3.32 (1.05–10.51)		1.49 (0.42–5.01)	

*HR* hazard ratio

### Adjusted Cox model

In the adjusted model including HIV status, stage of lymphoma, age and number of chemotherapy cycles received, the only statistically significant factor associated with death was late stage of lymphoma (AHR 5.45(*p* = 0.034, CI 1.14–26.06) [Table 2].

### Kaplan-Meier survival graph

**Fig. 1:**
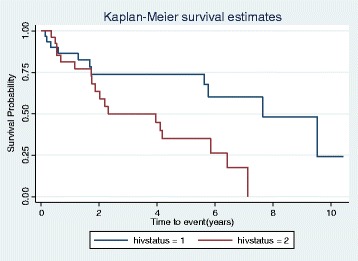
Kaplan Meier estimation of survival stratified by HIV status (HIV positive =red/2; HIV negative= black/1). Log-rank test for equality of survivor functions. Chi2 (1) = 6.32, P r >chi2 = 0.0120

The log-rank test for Kaplan-Meier graph were significantly different between those with and without HIV infection (*P* = 0.03). Median survival probability for HIV-infected group was 2.1 years while for HIV-uninfected group was 7.6 years. The 2-year survival probability after lymphoma diagnosis was 30% for HIV-infected and 74% for HIV-uninfected lymphoma patients (*p* = 0.012) [Fig. 1].

## Discussion

In this 10-year retrospective cohort study, mortality was higher (62.5%) among the HIV-positive group as most of them (75.0%) presented at late stages II and III [Table 1]. Late stage presentation at diagnosis amongst HIV-lymphoma patients was also reported in a similar study in Uganda by Bateganya et al. [31]. Only the stage of lymphoma at diagnosis was significantly associated with death after adjusting for HIV-status, stage, age and number of chemotherapy cycles received. In a similar 4-year retrospective study of 154 NHL patients by Bateganya et al., the same factors used in our study’s adjusted model were all significant in predicting death specifically in HIV-infected NHL patients not on ART [31]. But a similar study in the USA by Gopal et al. in a large cohort of NHL patients, showed only older age and HIV viremia to be predictive of survival [26]. Small sample size of lymphoma patients in this study could have accounted for stage being the only significant factor predictive of death.

Most HIV-infected patients (62%) in this study received lower cumulative cycles of chemotherapy (<4 cycles) within a year after initiation of lymphoma treatment. This could be due to inability to purchase chemotherapy drugs as this competes with other drugs the patients need to buy. Also is the fact that they have more probability of death (88%) within the first year period after initiation of lymphoma chemotherapy as observed from the Kaplan Meier graph [Fig. 1]. Only 2/40(5.0%) patients were lost to follow-up in this study, thus was not statistically significant.

The significant difference (*p* = 0.03) in the probability of survival observed between the HIV-infected and HIV-uninfected lymphoma groups in this study was similar to studies reported in the USA and Africa but contrary to studies from Europe which showed probability of survival in the HIV-infected group equating that in the HIV-uninfected group [23–27, 31–33]. Regional variation in the demographics of these populations, different treatment modalities and variability in the biology of lymphoma sub-types could account for this difference. The difference in probability of mortality between the two groups becomes wider after two years follow-up in this study likely because of high tumor recurrence in the HIV group (not measured in this study) coupled with increased financial burden in these patients with inability to cope with high cost of drugs as duration of follow up increases.

Management of this sub-population should therefore be geared towards early detection of disease and early institution of both chemotherapy and ART. Chemotherapy and other support drugs should be made available and affordable for patients to access easily. The current health insurance program in Nigeria should be expanded to cover cost of chemotherapeutic agents so as to reduce mortality in these patients. Government should as a minimum, upgrade certain hospitals in the country into a status of “centers of excellence for cancer care” equipped with sophisticated equipment for cancer diagnosis and treatment.

Limitations of this study includes use of secondary observational data, small cohort size, lack of information on actual treatment costs and inability to collect other outcome information such as presence of recurrence of lymphoma which might explain survival differences.

Despite these limitations, our study has several strengths. To our knowledge, this is the first study to determine mortality outcomes of HIV-lymphoma in our sub-region and to identify likely contributory factors. Results of this study could serve as initial findings for replication in other regions of the country and could stimulate future larger prospective studies as we continue to unravel the biology of lymphomas associated with HIV infected patients in the current ART era.

## Conclusion

Our findings suggest that HIV infection negatively impacts mortality after lymphoma diagnosis with stage at presentation being a significant determinant of survival in Jos, Nigeria. HIV-associated lymphoma in the ART era continues to contribute to morbidity and high mortality due to increased incidence, biologic heterogeneity and underlying virologic/immunologic factors. With unprecedented improvement in access to ART courtesy of the US President’s Emergency Plan for AIDS relief (PEPFAR) project in Nigeria, improved immunity has resulted in an aging HIV population. Future research should be done to better understand the biology of this highly heterogeneous tumor. The research should also be to identify key unique risk factors in this sub-population with renewed emphasis on individualized management to reduce mortality.

## Appendix

### A. Ann Arbor staging

Stage I indicates that the cancer is located in a single region, usually one lymph node and the surrounding area. Stage I often will not have outward symptoms.

Stage II indicates that the cancer is located in two separate regions, an affected lymph node or organ and a second affected area, and that both affected areas are confined to one side of the diaphragm—that is, both are above the diaphragm, or both are below the diaphragm.

Stage III indicates that the cancer has spread to both sides of the diaphragm, including one organ or area near the lymph nodes or the spleen.

Stage IV indicates diffuse or disseminated involvement of one or more extra-lymphatic organs, including any involvement of the liver, bone marrow, or nodular involvement of the lungs.

#### Modifier

A or B: the absence of constitutional (B-type) symptoms is denoted by adding an “A” to the stage; the presence is denoted by adding a “B” to the stage.

S: is used if the disease has spread to the spleen.

E: is used if the disease is “extra nodal” (not in the lymph nodes) or has spread from lymph nodes to adjacent tissue.

X: is used if the largest deposit is >10 cm large (“bulky disease”), or whether the mediastinum is wider than 1⁄3 of the chest on a chest X- ray.

### B. W.H.O. classification of lymphoma (2008)

Hodgkin.

Non-Hodgkin (mature B-cell, mature T-cell, NK-cell).
