# Erratum to: Outcome of HIV-associated lymphoma in a resource-limited setting of Jos, Nigeria

**DOI:** 10.1186/s13027-017-0149-2

**Published:** 2017-06-26

**Authors:** Olugbenga Akindele Silas, Chad J. Achenbach, Lifang Hou, Robert L. Murphy, Julie O. Egesie, Solomon A. Sagay, Oche O. Agbaji, Patricia A. Agaba, Jonah Musa, Agabus N. Manasseh, Ezra D. Jatau, Ayuba M. Dauda, Maxwell O. Akanbi, Barnabas M. Mandong

**Affiliations:** 1 Pathology Department, Faculty of Medical Sciences, University of Jos/Jos University Teaching Hospital, Jos, Plateau State Nigeria; 2 Feinberg School of Medicine, Department of Medicine, Northwestern University and Center for Global Health, Chicago, IL USA; 3 Hematology Department Faculty of Medical Sciences, University of Jos/Jos University Teaching Hospital, Jos, Plateau State Nigeria; 4 Department of Obstetrics and Gynecology Faculty of Medical Sciences, University of Jos/Jos University Teaching Hospital, Jos, Plateau State Nigeria; 5 Department of Internal Medicine Faculty of Medical Sciences, University of Jos/Jos University Teaching Hospital, Jos, Plateau State Nigeria; 6 Department of Family Medicine Faculty of Medical Sciences, University of Jos/Jos University Teaching Hospital, Jos, Plateau State Nigeria

## Erratum

After publication of the original article [1], the corresponding author noticed Patricia A. Agaba’s name was incorrectly included as Patricia E. Agaba in the author list. The correct presentation of the author name can be found in the author list of this erratum.

## References

[1] Silas OA, Achenbach CJ, Hou L (2017). Outcome of HIV-associated lymphoma in a resource-limited setting of Jos, Nigeria. Infectious Agents and Cancer.

